# Genomic prediction with epistasis models: on the marker-coding-dependent performance of the extended GBLUP and properties of the categorical epistasis model (CE)

**DOI:** 10.1186/s12859-016-1439-1

**Published:** 2017-01-03

**Authors:** Johannes W. R. Martini, Ning Gao, Diercles F. Cardoso, Valentin Wimmer, Malena Erbe, Rodolfo J. C. Cantet, Henner Simianer

**Affiliations:** 1 Department of Animal Sciences, Georg-August University, Albrecht Thaer-Weg 3, Göttingen, Germany; 2 National Engineering Research Center for Breeding Swine Industry, Guangdong Provincial Key Lab of Agro-animal Genomics and Molecular Breeding, College of Animal Science, South China Agricultural University, Guangzhou, China; 3 Departamento de Zootecnia, São Paulo State University, São Paulo, Brazil; 4 KWS SAAT SE, Einbeck, Germany; 5 Institute for Animal Breeding, Bavarian State Research Centre for Agriculture, Grub, Germany; 6 Department of Animal Production, University of Buenos Aires, INPA-CONICET, Buenos Aires, Argentina

**Keywords:** Genomic prediction, Epistasis model, Interaction

## Abstract

**Background:**

Epistasis marker effect models incorporating products of marker values as predictor variables in a linear regression approach (extended GBLUP, EGBLUP) have been assessed as potentially beneficial for genomic prediction, but their performance depends on marker coding. Although this fact has been recognized in literature, the nature of the problem has not been thoroughly investigated so far.

**Results:**

We illustrate how the choice of marker coding implicitly specifies the model of how effects of certain allele combinations at different loci contribute to the phenotype, and investigate coding-dependent properties of EGBLUP. Moreover, we discuss an alternative categorical epistasis model (CE) eliminating undesired properties of EGBLUP and show that the CE model can improve predictive ability. Finally, we demonstrate that the coding-dependent performance of EGBLUP offers the possibility to incorporate prior experimental information into the prediction method by adapting the coding to already available phenotypic records on other traits.

**Conclusion:**

Based on our results, for EGBLUP, a symmetric coding {−1,1} or {−1,0,1} should be preferred, whereas a standardization using allele frequencies should be avoided. Moreover, CE can be a valuable alternative since it does not possess the undesired theoretical properties of EGBLUP. However, which model performs best will depend on characteristics of the data and available prior information. Data from previous experiments can for instance be incorporated into the marker coding of EGBLUP.

**Electronic supplementary material:**

The online version of this article (doi:10.1186/s12859-016-1439-1) contains supplementary material, which is available to authorized users.

## Background

Genomic prediction aims at forecasting qualitative or quantitative properties of individuals based on known genetic information. The genetic information can for instance be given by single-nucleotide-polymorphisms (SNPs) or other kinds of genetic data of individual animals, plant lines or humans. Applied to animals and plants, genomic prediction is of central importance for breeding within the concept of *genomic selection* [1, 2]. Moreover, genomic prediction can also be used in medicine or epidemiology for risk assessment or prevalence studies of (partially) genetically determined diseases (e.g. [3]). One of the standard approaches for genomic prediction of quantitative traits is based on a linear regression model in which the phenotype is described by a linear function of the genotypic markers. In more detail, the standard additive linear model is defined by the equation

1 $$  \mathbf{y} = \mathbf{1}\mu + \mathbf{M} \boldsymbol{\beta} + \boldsymbol{\epsilon}  $$

where **y** is the *n*×1 vector of phenotypes of the *n* individuals, **1** the *n*×1 vector with each entry equal to 1, *μ* the fixed effect and **M** the *n*×*p* matrix giving the *p* marker values of the *n* individuals. Moreover, ***β*** is the *p*×1 vector of unknown marker effects and ***ε*** a random *n*×1 error vector with $\epsilon _{i} {\overset {i.i.d.}{\sim }}\mathcal {N}(0,\sigma _{\epsilon }^{2})$. Since the number of markers *p* is typically much larger than the number of individuals *n*, the additional assumption that $\beta _{j} \overset {i.i.d.}{\sim } \mathcal {N}(0,\sigma _{\beta }^{2})$ is usually made (and all random terms together are considered as stochastically independent). In particular, using an approach of maximizing the density of a certain distribution [4], this assumption allows us to determine the penalizing weight in a Ridge Regression approach which is known as *ridge regression best linear unbiased prediction* (RRBLUP) and which is fully equivalent to its relationship matrix-based counterpart *genomic best linear unbiased prediction* (GBLUP)^1^ [5, 6]. The answer to the question which type of marker coding is appropriate in **M** depends on the combination of the type of genotypic marker and ploidy of the organism dealt with. For instance, if haploid organisms are considered or presence/absence markers are used, a possible coding for the *j*-th marker value of the *i*-th individual *M*_*i,j*_ is the set {0,1}. Counting the occurrence of an allele of a diploid organism, the sets {0,1,2} or {−1,0,1}, or rescaled variants can be used. If the marker effects ***β*** and the fixed effect *μ* are predicted/estimated as $\boldsymbol {\hat {\beta }}$ and $\hat {\mu }$ on the basis of a training set, the expected phenotypes of individuals from a test set, which were not used to determine $\boldsymbol {\hat {\beta }}$ and $\hat {\mu }$, can be predicted by using their marker information in Eq. (1) with $\hat {\mu },\boldsymbol {\hat {\beta }}$. We will call the difference between the predicted expected phenotype and the estimated fixed effect the predicted *genetic value*. For the purely additive model of Eq. (1) and a diploid organism with possible genotypes *aa*, *aA* and *AA* for locus *j*, the choice of how to translate these possibilities into numbers was reported not to affect the predictive ability notably, as long as the difference between the coding of *aa* and *aA* is the same as between *aA* and *AA* and equal for all markers [5, 7–9]. However, an extension of the additive model, which we call the *extended* GBLUP model (EGBLUP) [10, 11]

2 $$  y_{i} = \mu + \sum\limits_{j=1}^{p} M_{i,j} \beta_{j} + \sum\limits_{k=1}^{p}\sum\limits_{j=k}^{p} M_{i,j}M_{i,k} h_{j,k} + \epsilon_{i},  $$

has been shown to exhibit strong coding dependent performance [12, 13]. Here, $h_{j,k}\overset {i.i.d.}{\sim } \mathcal {N}\left (0,{\sigma ^{2}_{h}}\right)$ is the pairwise interaction effect of markers *j* and *k* and all other variables as previously defined (all terms stochastically independent). Compared to Eq. (1), this model additionally incorporates pairwise products of marker values as predictor variables and thus allows us to model interactions between markers. Moreover, the interaction of a marker with itself gives a possibility to model dominance effects (see e.g. [11, 14–16]). The epistasis model of Eq. (2) and some variations with restrictions on which markers can interact have been the main object of investigation in several publications and models incorporating epistasis have been viewed as potentially beneficial for the prediction of complex traits [10, 11, 17–19], but a marker coding dependent performance was observed [12, 13].

In this work, we investigate how the marker coding specifies the effect model for markers with two or three possible values and show how we can find the marker coding for an a priori specified model. We discuss advantages and disadvantages of different coding methods and investigate properties of alternative linear models based on categorical instead of numerical dosage variables. In particular, we show how to represent these models as genomic relationship matrices. Finally, we compare the predictive abilities of different epistasis models on simulated and publicly available data sets and demonstrate a way of using the coding-dependent performance of EGBLUP to incorporate prior information.

## Methods

### Data sets used for assessing predictive ability

#### Simulated data

A population with 10 000 bi-allelic markers spread across five chromosomes was simulated, using the QMSim software [20]. The size of the first chromosome was 140 centimorgan (cM) with 3 500 markers. Chromosomes 2 to 5 had a size of 110 cM (2 750 markers), 80 cM (2 000 markers), 50 cM (1 250 markers) and 20 cM (500 markers), receptively. In order to allow mutations and linkage disequilibrium establishment, a historical population was simulated with 5 000 individuals (2 500 males and 2 500 females) with random mating for 1 000 generations with constant population size and with a replacement rate of 0.2 for males and females. Then the population size was reduced to 1 000 individuals for 20 additional generations (generation 1 001 to 1 020). The simulated mutation rate was 2.5·10^−5^.

We used this simulated genotypes as basis and modeled three different types of genetic architecture (purely additive, purely dominant and purely epistatic), each with a varying number of quantitative trait loci (QTL) on top. We chose these types of genetic architecture, without additive effects in the dominance and epistasis scenarios, to make the three scenarios as different as possible. To model the phenotype, out of the 10 000 markers, 200 were drawn randomly from each of the five chromosomes to define in total 1 000 QTL for additive or dominance effects. For the purely additive scenario, the 1 000 additive effects were drawn independently from a $\mathcal {N}(0,1)$ distribution. For the first additive trait A1, 10 out of the 1 000 QTL were drawn and the genetic values of all individuals were calculated according to the effects of these 10 loci. To define a broad sense heritability of 0.8, the genetic values were standardized to mean 0 and variance 1 and individual errors were drawn from a $\mathcal {N}(0,0.25)$ distribution. Having added these individual errors to the genetic values, these phenotypes were again standardized to mean 0 and variance 1. For the second trait A2, additional 90 QTL were drawn from the initial 1 000 to give in total 100 QTL for this trait including the QTL of trait A1 with their corresponding effects. Analogously, for A3, all initially drawn 1 000 QTL were used. The standardization procedure was identical to the one previously described for A1. For the comparison of genomic prediction with different relationship models, these 1 000 markers were removed. The relationship matrices were based on the remaining 9 000 markers.

For the dominance scenario D1 (10 QTL), D2 (100 QTL) and D3 (1 000 QTL), we used the same QTL positions as for A1, A2, and A3, respectively, but simulated $\mathcal {N}(0,1)$-distributed dominance effects. The standardization procedure to a broad sense heritability of 0.8 was carried out as described before.

For the epistasis traits E1, E2 and E3, 1 000, 10 000 or 100 000 pairs of markers were drawn randomly and for each draw, one of the nine possible configurations of the pair was randomly chosen to have an $\mathcal {N}(0,1)$-distributed effect. For instance, having drawn the marker pair *j,k*, only the configuration (*M*_*i,j*_,*M*_*i,k*_)=(0,2) was chosen to have an effect, which again was drawn randomly. This was done independently for each trait, which means trait E2 does not necessarily share causal combinations of markers with trait E1. The phenotypes were standardized as described above. Note, that the markers involved in causal combinations were not removed here, since in expectation, every marker is somehow involved in the phenotype of trait E2 and E3.

We repeated this whole procedure, including the simulation of the genotypes, 20 times and compared the different models by their average predictive ability across the 20 repetitions. The simulated data can be found in Additional file 1 of this publication.

#### Wheat data

The wheat data which we used to compare different methods was published by Crossa et al. [21]. The 1279 DArT markers of 599 CIMMYT inbred wheat lines indicate whether a certain allele is present (1) or not (0). The phenotypic data describes standardized records of grain yield under four environmental conditions.

#### Mouse data

The mouse data set we used was published and described by Solberg et al. [22] and Valdar et al. [23], and was downloaded from the corresponding website of the Wellcome Trust Centre for Human Genetics. The physical map of single nucleotide polymorphisms (SNPs) was updated to the latest version of the mouse genome (*Mus musculus*, assembly GRCm38.p4) with the biomaRt R package [24, 25]. Only SNPs mapped to the GRCm38.p4 were used for further analysis. For the remaining markers, the ratio of missing marker values was rather low (0.33%) and we performed a random imputation. The nucleotide coded genotypes were translated to a {0,1,2} coding, where 0 and 2 denote the two homozygous and 1 the heterozygous genotype. SNPs with minor allele frequency (MAF) smaller than 0.01 were excluded from the dataset. Imputation, recoding, and quality control of genotypes were carried out with the synbreed R package simultaneously [26]. A number of 9265 SNPs remained in the dataset for further analysis. We only used individuals with available records for all considered traits for further analysis, which reduced the number of individuals to 1 298. We focused on the provided pre-corrected residuals of 13 traits from which fixed effects of trait-specific relevant covariates such as sex, season, month, have already been subtracted. A detailed description of the traits can be found on the corresponding sites of the UCL. Moreover, the data resulting from quality control and filtering as well as the corrected phenotypes of the traits we used can be found in Additional file 1.

### Genomic relationship based prediction and assessment of predictive ability

We used an approach based on relationship matrices for genomic prediction. The underlying concept of this approach is the equivalence of marker effect-based and genomic relationship-based prediction ([5, 10, 11]). Given the respective relationship matrix, the prediction is performed by Eq. (3) (for a derivation of this equation see the supporting information of [11]):

3 $$ \begin{aligned} {}\left(\begin{array}{c} \hat{\mathbf{g}}_{train}\\ \hat{\mathbf{g}}_{test} \end{array}\right) & =\left[\mathbf{T}_{train} - s^{-1} \left(\begin{array}{cc} \mathbf{J}_{s \times s} & 0 \\ 0& 0 \end{array}\right)\right. \\ & \quad \left. + \sigma_{\epsilon}^{2} \left(\frac{1}{\sigma^{2}_{\beta}} \mathbf{G}^{-1}\right) \right]^{-1} \!\left(\! \left(\begin{array}{c} \mathbf{y}_{train} \\ 0 \end{array}\right) \!- \left(\begin{array}{c} \mathbf{1}_{s} \bar{y}_{train}\\ 0 \end{array} \right)\! \right) \end{aligned}  $$

The matrix **G** is the central object denoting the genomic relationship matrix of the respective model. The variables $\hat {\mathbf {g}}_{i}$ are the predicted genetic values (expected phenotype minus the fixed effect $\hat {\mu }$) of the respective set (training or test set). Moreover, *s* is the number of genotypes in the training set, **1**_*s*_ is the vector of length *s* with each entry equal to 1, **J**_*s*×*s*_ is the analogous *s*×*s* matrix with each entry equal to 1 and $\bar {y}_{train}$ is the empirical mean of the training set. Here, **T**_*train*_ denotes the diagonal matrix of dimension *n* with 0 on the diagonal at the positions of the test set genotypes, and 1 for the training set individuals.

To assess the predictive ability of different models, we chose a test set consisting of ∼ 10*%* of the total number of individuals (100, 60, or 130 for the simulated, the wheat and the mouse data, respectively). We then used the remaining individuals as a training set and predicted the genetic values for all individuals using Eq. (3). The variance components $\sigma _{\epsilon }^{2}$ and $\sigma _{\beta }^{2}$ were estimated from the training set using version 3.1 of the R package EMMREML [27]. The relationship matrix relating the genotypes of the training set was used to estimate the variance components based on the phenotypes of the training set only. The variance components were then used with the complete relationship matrix for the prediction of the genetic values of all individuals in Eq. (3). This procedure was repeated 200 times, with independently drawn test sets. The average correlation *r* between observed and predicted mean phenotypes of the test set was used as a measure of predictive ability. A description of how the different effect models can be translated into relationship matrices is given in the results. For the Gaussian kernel, we used the bandwidth parameter $b=2q_{0.5}^{-1}$, with *q*_0.5_ the median of all squared Euclidean distances between the individuals of the respective data. For the simulated data which consisted of 20 independent data sets, we present the average predictive ability and the average standard error of the mean. For the wheat and the mouse data, we used Tukey’s ‘Honest Significant Difference’ test to contrast the performance of the different prediction methods (TukeyHSD() and lm() of R [28]).

### Incorporation of prior information by marker coding

As described above, the data we used offers records of different traits or trait ×environment combinations of the same individuals. We will illustrate that the coding-dependent performance of EGBLUP can also be used to incorporate a priori information into the model by choosing the coding for each interaction with already provided data and by using the corresponding relationship matrix for prediction under altered environmental conditions or for a correlated trait. We used for the wheat data the following procedure:

1. We predicted all the interactions $\hat {h}_{k,l}$ for a given trait in a given environment, under the use of the {0,1} coding originally provided by Crossa et al. [21] (as described by Martini et al. [11]).
2. We changed the “orientation” of all markers at once by substituting 0 by 1, and 1 by 0 and predicted all interactions $\tilde {h}_{k,l}$ under the use of the altered coding.
3. If the ratio of $ \left |\frac {\hat {h}_{k,l}}{\tilde {h}_{k,l}} \right |$ was greater than or equal to 1, we assumed that the original orientation provided by the data set describes the respective interaction better than the alternative coding.
4. We then calculated a relationship matrix for each interaction individually by
  $$\mathbf{G}_{k,l} = \mathbf{\left(M_{\bullet, k} M_{\bullet, k}^{\prime} \right) \circ \left(M_{\bullet, l} M_{\bullet, l}^{\prime} \right)} $$
  with **M**_∙,*k*_ denoting the *n*×1 vector of marker data of locus *k* for all individuals in the respective coding which seems to fit the interaction better according to 3) (see [11, 29]). Here, ∘ denotes the Hadamard product.
5. The overall relationship matrix was then defined by $\mathbf {G}= \sum \limits _{k=1}^{p} \sum \limits _{l \geq k}^{p}\mathbf {G}_{k,l}$.

We used the data of each environment to calculate an optimally coded relationship matrix for this environment, which was used afterwards for predicting phenotypes in the other environments. The underlying heuristic of step 3) is that a small effect means that the interaction is less important in the respective coding. If the underlying effect model defined by the coding does not capture the data structure, the estimated effect should be close to zero. However, if the effect of a combination is important to describe the phenotype distribution, a larger effect should be assigned (see also Example 1, where the estimated effect is 0, if the underlying parameterization cannot describe the present effect distribution).

For the mouse data, we used the 13 considered traits to construct a relationship matrix for each of them. Each relationship matrix was afterwards used for prediction within the data of the twelve other traits. The two different codings which were compared here, were the {0,1,2} coding based on the imputed originally provided data and its inverted version with 0 and 2 permuted.

## Results

In the following, we will highlight aspects of the behavior of the additive effect model of Eq. (1) when the marker coding is altered. These properties of the additive model will afterwards be compared to those of the epistasis model of Eq. (2).

All relationship matrices will be assumed to be positive definite and thus invertible. Mathematical derivations of the illustrated properties can be found in Additional file 2.

### Properties of GBLUP

We start with the effect of translations of the coding, that is the addition of a number *p*_*j*_ to the initially chosen marker coding of marker *j*.

#### **Property 1**

(Translation-invariance of GBLUP) Let **P** denote a vector whose entries give the arbitrary translations *p*_*j*_ of the coding of the locus *j*. Moreover, let the ratio of $\sigma _{\epsilon }^{2}$ and $\sigma _{\beta }^{2}$ be known and unchanged if the marker coding is translated. Let $\boldsymbol {\hat {\beta }}$ and $\hat {\mu }$ denote the predicted / estimated quantities if the initial coding **M** is used in the Mixed Model Equation approach of Eq. (1) and let $\boldsymbol {\tilde {\beta }}$ and $\tilde {\mu }$ denote the corresponding quantities if the translation $\tilde {\mathbf {M}}:=\mathbf {M}-\mathbf {1}\mathbf {P^{\prime }}$ is used instead of **M**. Then the following statements hold:

- $\tilde {\mu }=\hat {\mu } + \mathbf {P^{\prime }} \boldsymbol {\hat {\beta }}$
- $\boldsymbol {\tilde {\beta }}=\boldsymbol {\hat {\beta }}$
- The prediction of the expected phenotype of each genotype is independent of whether **M** or $\tilde {\mathbf {M}}$ is used.

The statement of Property 1 has already been discussed in literature [5, 7–9], and we will present a mathematical derivation based on the Mixed Model Equations in Additional file 2. The proof will be a blueprint for the derivation of other properties based on the Mixed Model Equations which can also be found in Additional file 2. Descriptively, we can see the presented invariance with respect to translations the following way: If we change the coding to $\tilde {\mathbf {M}}:=\mathbf {M}-\mathbf {1P^{\prime }}$, then $\tilde {\mathbf {M}}$, $\tilde {\mu }:=\hat {\mu } + \mathbf {P^{\prime }} \boldsymbol {\hat {\beta }}$ and $\boldsymbol {\tilde {\beta }}:=\boldsymbol {\hat {\beta }}$ will fit the phenotypes the same way as **M**, $\hat {\mu }$ and $\boldsymbol {\hat {\beta }}$ do. Thus, the prediction of the marker effects and consequently the prediction of the expected phenotypes of individuals will not be affected by the change of coding as long as the method of evaluating the “goodness of fit”, that is the penalizing weight in a Ridge Regression approach remains unchanged. For this reason, it is important to note here that we made the precondition that the ratio of the variance components, which defines the penalty for effect size, will not be changed. This guarantees that the method of how to quantify the “goodness of fit” remains the same. In practice this may not exactly be the case if the vector **P** has non-identical entries, that is if the translation of the coding is not equal for all loci, since the variance components are usually estimated from the same data and the translation may have an effect on this estimation. However, this effect has been assessed as being negligible in practice [9]. To assess this problem from a theoretical point of view, without preconditions on the changes of ${\sigma ^{2}_{i}}$, the method for determining the variance components has to be taken into account to see whether a change in the marker coding has an influence on the ratio of the determined variance components. The next property considers the effect of rescaling the given marker coding.

#### **Property 2**

(Scaling invariance of GBLUP) Let $\boldsymbol {\hat {\beta }}$, $\hat {\mu }$, $\boldsymbol {\tilde {\beta }}$ and $\tilde {\mu }$ denote the quantities as defined in Property 1 with $\tilde {\mathbf {M}}:=c \mathbf {M}$ for a *c*≠0. Moreover, let $\sigma _{\epsilon }^{2}$ and $\sigma _{\beta }^{2}$ for **M** be known and let the variance components used for the Ridge Regression approach based on $\tilde {\mathbf {M}}$ fulfill $\frac {\tilde {\sigma }_{\epsilon }^{2}}{\tilde {\sigma }_{\beta }^{2}}=c^{2}\frac {\sigma ^{2}_{\epsilon }}{\sigma _{\beta }^{2}}$. Then the following statements hold:

- $\tilde {\mu }=\hat {\mu } $
- $\boldsymbol {\tilde {\beta }}=c^{-1}\boldsymbol {\hat {\beta }}$
- The prediction of the expected phenotype of each genotype is independent of whether **M** or $\tilde {\mathbf {M}}$ is used.

An important aspect of Property 2 is the precondition that the ratio of the variance components is adapted. In practice, when $\sigma _{\beta }^{2}$ is estimated, we can assume that this circumstance will approximately be given, however, we have to highlight again that this also depends on the method of how the variance components are determined.

### Epistasis models of shape of Eq. (2)

The full EGBLUP model of Eq. (2) adds interaction terms of shape *h*_*j,k*_ *M*_*i,j*_ *M*_*i,k*_ to the additive model of Eq. (1). We will focus on the properties of these additional terms in the following. Evidently, the product structure of the additional covariates generates a dependence of the underlying effect model on the marker coding. In particular, the genotype coded as zero has a special role. If *M*_*i,j*_ equals zero, the whole term *h*_*j,k*_ *M*_*i,j*_ *M*_*i,k*_ will be equal to zero, independently of the values of *h*_*j,k*_ and *M*_*i,k*_. Thus, the model has the implicit assumption that a certain set of combinations do not interact. The marker coding decides which interactions are different from zero a priori and which combinations are clustered. For instance, for the coding {−1,0,1} for the genotypes {*aa,aA,AA*} of a diploid organism, any interaction with a heterozygous locus will be zero, whereas the interactions with the homozygous locus *aa* will be zero if the coding {0,1,2} is used. Table 1 illustrates the differences of the two different standard codings ({−1,0,1} vs. {0,1,2}). Here we see that the marker coding {0,1,2} implies that the effect is monotonously increasing (or decreasing if *h*_*j,k*_ is negative) with the distance from the origin, whereas the coding {−1,0,1} gives a different topology by only giving weight to the double homozygous. It is not obvious which coding is to be preferred and which reasonable assumptions on the effect of pairs can be made. In the following, we will discuss theoretical properties of the model induced by the marker coding.

**Table 1:** Comparison of the interaction effects which are given implicitly by the marker coding {−1,0,1} (left) and {0,1,2} (right) in the interaction terms of EGBLUP. Each entry has to be multiplied with the interaction effect *h*_*j,k*_

	

As a first important observation, we note that the codings {−1,0,1} and {0,1,2} are translations of each other. Their very different interaction effect topologies illustrate that the epistasis model is not invariant with respect to translations. This fact that translations modify the model also makes obvious that by subtracting the matrix **1** **P** ^′^ with **P** containing the allele frequencies of the respective marker, which is the standard normalization in the additive model [6], we will change the coding for the markers according to their frequencies and thus implicitly use different effect models for each pair of loci. We do not see a theoretical basis for this discrimination in an infinitesimal model without additional prior knowledge and therefore will consider mainly models which treat markers equally. Moreover, as gene frequencies are sometimes poorly estimated and very influential, avoiding their use seems to be appealing.

As illustrated, the epistasis model is not invariant with respect to translations, but we show now that the previously described invariance with respect to rescaling persists also for the epistatis model.

#### **Property 3**

(Scaling invariance of EGBLUP) Let $\boldsymbol {\hat {\beta }}$, $\hat {\mu }$, $\boldsymbol {\tilde {\beta }}$ and $\tilde {\mu }$ denote the quantities as defined in Property 1 with $\tilde {\mathbf {M}}:=c \mathbf {M}$ for a *c*≠0. Moreover, let $\boldsymbol {\hat {h}}$ and $\boldsymbol {\tilde {h}}$ denote the corresponding predictions for the interaction effects. Let $\sigma _{\epsilon }^{2}$, $\sigma _{\beta }^{2}$, ${\sigma _{h}^{2}}$ for **M** be known and let the variance components used for the Ridge Regression approach based on $\tilde {\mathbf {M}}$ fulfill $\frac {\tilde {\sigma }_{\epsilon }^{2}}{\tilde {\sigma }_{\beta }^{2}}=c^{2}\frac {\sigma ^{2}_{\epsilon }}{\sigma _{\beta }^{2}}$ and $\frac {\tilde {\sigma }_{\epsilon }^{2}}{\tilde {\sigma }_{h}^{2}}=c^{4}\frac {\sigma ^{2}_{\epsilon }}{{\sigma _{h}^{2}}}$. Then the following statements hold:

- $\tilde {\mu }=\hat {\mu } $
- $\boldsymbol {\tilde {\beta }}=c^{-1}\boldsymbol {\hat {\beta }}$
- $\boldsymbol {\tilde {h}}=c^{-2}\boldsymbol {\hat {h}} $
- The prediction of the expected phenotype of each genotype is independent of whether **M** or $\tilde {\mathbf {M}}$ is used.

A formal derivation of this property based on the Mixed Model Equations can be found in the Additional file 2, but the statements are also plausible if we follow the descriptive argumentation for the invariance of the additive model: If $\hat {\mu }$, $\boldsymbol {\hat {\beta }}$ and $\boldsymbol {\hat {h}}$ fit the phenotypic data best when marker matrix **M** is used, $c^{-1}\boldsymbol {\hat {\beta }}$ and $c^{-2}\boldsymbol {\hat {h}}$ will fit the phenotypic data the same way if **M** is substituted by $\tilde {\mathbf {M}}$ in Eq. (2) (for any constant *c*≠0). The important precondition is that the penalizing weight, which defines which fit is “best”, is adapted. A question that might come up in the context of Properties 2 and 3 is whether we could also multiply each coding for locus *j* with its own constant *c*_*j*_≠0, similar to what we had for Property 1 and vector **P**. A problem that will appear here is that the variance of the marker effects will not be changed uniformly and thus, we cannot simply adapt the variance components to cancel the impact of rescaling. An individual rescaling and thus weighting of each marker [30], as well as a completely individual coding of each genotype of each locus, without the side conditions that the differences in the coding of the heterozygous and the two homozygous genotypes are identical across all loci or at least symmetric for each locus [12, 13], indeed has an impact on the predictive ability of the models, in particular also on that of GBLUP. However, the variance components ${\sigma _{i}^{2}}$ can be globally adapted to cancel the impact of a non-uniform rescaling of the marker coding, in case that some columns of **M** are multiplied with *c* and the others with −*c* (due to the assumption of all effects being symmetrically distributed around mean zero). An adapted sign of the effects also allows the predicted effect model to remain unchanged.

**Permuting the role of the alleles at locus** ***j*** **.** Let locus *j* have the possible allele configurations *aa*, *aA* and *AA*. The prediction performance of GBLUP is unaffected by the choice of whether the allele variant *a* or *A* is counted, since we can express a permutation of the initial coding {0,1,2} by a translation by −2 and a multiplication of the coding by −1.

Obviously, this argumentation cannot be used for the epistasis model, since we do not have the possibility to translate the marker coding. This fact raises the question under which circumstances the epistasis EGBLUP model is unaffected by a permutation of the role of the allele variants.

#### **Property 4**

(Symmetric role of the alleles in EGBLUP) Let us consider locus *j* with alleles *a* and *A* and locus *k* with alleles *b* and *B* (of a diploid organism). Let us use the same coding for both loci and let the three variants of *aa*, *aA* and *AA* be coded by three different numbers *M*_*aa*_<*M*_*aA*_<*M*_*AA*_ (or *M*_*aa*_>*M*_*aA*_>*M*_*AA*_). The only coding for the epistasis terms, whose corresponding effect model on the tuples

$$ \left\{ (j,k) | j \in \{aa,aA,AA\}, k \in\{bb,bB,BB\} \right \} $$

is invariant with respect to a permutation of the role of allele *a* and *A* satisfies −*M*_*aa*_=*M*_*AA*_ and *M*_*aA*_=0. Analogously, for markers with only two possible values, the coding has to satisfy −*M*_*a*_=*M*_*A*_.

Property 4 is of central theoretical importance since it implies that the only coding for {0,1} marker in EGBLUP, which is invariant with respect to a permutation of the meaning of 0 and 1 is the coding {−*c,c*} (*c*≠0). Moreover, if EGBLUP shall possess this reasonable property for markers with three possible values, we have to use the coding {−*c*,0,*c*}. We will give an example to illustrate why this property is important for determining marker effects and thus why it may also be important for the overall predictive ability of the model.

#### **Example 1**

(Marker effects and quadratic loss) Let us consider markers with two possible variants and let us assume that for each pair of markers, the correct underlying weights of the combinations is given by a coding as {0,1}. We use a {0,1} coding, but we do not know which variants of the two loci have to be coded as 1 to capture the real effect distribution. We assume that we decide which allele is coded as zero, by drawing independently from a Bernoulli-distribution with *p*=0.5 for each marker. To see how good the real underlying weight distribution is captured, we measure the quadratic loss between the best possible fit and the real underlying weights. Let the coding

4 $$ \begin{array}{c | c | c} & a & A \\ b & 0& 0 \\ B & 0 & 1\\ \end{array}  $$

be the correct underlying effect distribution, with the corresponding underlying interaction effect equal to 1 (the problem remains the same if the underlying interaction effect is multiplied with any number *c*≠0). With a probability of 0.25, we will code both markers *j* and *k* correctly and minimize the distance to zero by predicting $\hat {h}_{j,k}=1$. However, with a probability of 0.75, we will make a mistake and choose an incorrect orientation, which means an incorrect underlying parametric model, such as

5 $$ \begin{array}{c|c|c} & a & A \\ b & 1 \cdot \; h_{j,k} & 0 \\ B & 0 & 0\\ \end{array}  $$

In this situation, we can determine the optimally fitting interaction $\hat {h}_{j,k}$, which describes the distribution of Eq. (4) best, when model Eq. (5) is used, by minimizing the quadratic Euclidean distance between both effect distributions. In more detail, using a minimal quadratic loss means we have to find an $\hat {h}_{j,k}$ which minimizes the quadratic distance between the matrices of Eq. (4) and Eq. (5):

6 $$ (1h_{j,k}-0)^{2}+(0-0)^{2}+(0-0)^{2}+(0-1)^{2}  $$

which is equal to

$$h_{j,k}^{2}+1. $$

Thus, the optimal $\hat {h}_{j,k}$ minimizing Eq. (6) is 0 and the expected quadratic loss when the right coding with unknown orientation is used, is 0.25·0+0.75·1=0.75.

Analogously, if we use the coding {−1,1} instead of Eq. (5), we will obtain the quadratic distance

$${}3(h_{j,k}-0)^{2} + (h_{j,k}-1)^{2} \qquad \text{or} \qquad 3(h_{j,k}-0)^{2} + (h_{j,k}+1)^{2} $$

each with probability 0.5, depending on whether −1 or +1 coincides with the 1 of the real underlying effects. Consequently, the minimum quadratic distance is 0.75 with probability 1, for $\hat {h}_{j,k}= \pm 0.25$. Thus, in this example, even though the coding {−1,1} specifies a model which is surely wrong, the average quadratic loss is equal to the situation in which we know the exact shape of the effect distribution but not its orientation. If the real underlying effect distribution deviates from the {0,1} coding of Eq. (4), the possibility to adapt the orientation might be even more important.

Example 1 illustrated that the expected quadratic loss of the estimated marker-pair weights is equal for the codings {−1,1} and {0,1} even in the case that the underlying effects are a version of the latter one but with unknown orientation. Moreover, we can observe the following: Let us assume that the real underlying interactions (*j,k*),(*j,l*) and (*k,l*) of the three loci *j,k,l* are described by certain {0,1}-codings, meaning that one certain configuration has an interaction effect but the others do not. Given the underlying effects, we can adapt the coding of *j,k* and *l* by considering the effects of the pairs (*j,k*),(*j,l*). However, then the effect distribution within the model is also determined for the pair (*k,l*), because the marker coding has already been fixed. This configuration does not necessarily describe the interaction of (*k,l*) well. This fact illustrates that due to the way of how interactions are incorporated into the model in EGBLUP, the model with an asymmetric coding lacks a full flexibility to adapt to any situation. This problem does not appear with the symmetric coding, since the model is independent of the decision which allele is coded as ±1. However, there are also good reasons for choosing other types of coding. Firstly, it is not clear whether the effect that we have illustrated on the level of marker effects and quadratic loss, also translates to the level of prediction of genetic values. In the latter approach, all effects are predicted simultaneously and thus errors of individual effects can cancel out in the sum. Secondly, from a biological point of view, the symmetric coding seems inadequate: Let us consider markers with two variants and let the two loci *j* and *k* have the possible variants *a,A* and *b,B*, respectively. The symmetric coding {−1,1} assigns the weight 1*h*_*j,k*_ to the combinations (*a,b*) and (*A,B*), meaning that the most distant genotypes, which do not share any allele, are treated as being equal in the model. Thus, overall, it is not clear which coding will be most appropriate in general. Especially in situations in which additional information on the nature of the marker or the biology of the trait is available, this information may be used to specify the effect model. In the next paragraph, we illustrate how much freedom the marker coding gives to specify the model.

**Finding the marker coding for an a priori specified model.** Let us consider a model with identical marker coding *M*_*aa*_, *M*_*aA*_ and *M*_*AA*_ for each locus. Then the weights in the model are given by

7 $$\begin{array}{@{}rcl@{}} a_{1,1}=M_{aa}^{2} & a_{1,2}=M_{aa}M_{aA} & a_{1,3}=M_{aa}M_{AA}\\ a_{2,2}=M_{aA}^{2} & a_{2,3}=M_{aA}M_{AA} & a_{3,3}=M_{AA}^{2}. \end{array} $$

If we want to predefine the weights *a*_*r,s*_ and calculate a corresponding coding, we see that not all choices of weights can be translated into a coding for the epistasis model of Eq. (2) since contradictions can arise. However, the following statement holds:

#### **Property 5**

Let three weights *a*_*r,s*_ of Eq. (7) which include the three variables *M*_*aa*_, *M*_*aA*_, *M*_*AA*_ in at least one weight *a*_*r,s*_ be given by arbitrary nonzero numbers. Then the marker codings as well as the other weights are determined up to their signs.

### Categorical effect models

In the following, we discuss categorical effect models in which we do not treat the marker data as numerical dosage, but as categorical variables. The goal is to build an epistasis model without the undesired properties of EGBLUP which have been described previously. We model the effects of allele *combinations* as being independently drawn from a Gaussian distribution with mean zero. For instance, for an additive marker effect model, the effects of *aa*, *aA* and *AA* are independently originating from the same distribution. For the analogous epistasis model, the effect of each combination of the alleles of two loci is drawn independently from the same distribution. We will introduce dummy {0,1} variables to indicate which allele configuration is present and thus inflate the number of variables in our model. The important fact to notice in this context is that we can use a relationship matrix approach for genomic prediction (see “Methods”) and thus do not need to handle the high number of variables. This procedure also reduces computation time compared to the effect based approach. All considered effects *β*_*j*_ of the variables are assumed to come from the same distribution: $\beta _{j}\overset {i.i.d.}{\sim } \mathcal {N}(0,\sigma _{\beta }^{2})$.

**Fig. 1:**
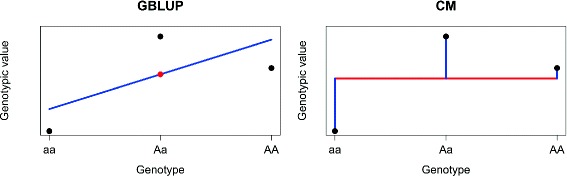
Comparison of the parametrization of the genotypic values in GBLUP and the categorical marker effect model CM: Black dots: genotypic values of the corresponding genotype of a certain locus. GBLUP parameterizes the genotypic values by a fixed effect (red dot) and a random effect determining the slope (*blue* line), whereas CM parameterizes by the fixed effect (*red* line) and independent random effects (*blue* lines) for each genotype

**A categorical marker effect model (CM)** The underlying concept of this model is to code the configurations *aa,aA,AA* of locus *j* as three different variables. The effect of each genotype is estimated on its own. The assumption of a constant allele substitution effect, that is that the effect of *AA* equals twice the effect of *A*, which is made in the additive numerical GBLUP model, is not made here (see Fig. 1). We translate the genotypes (*aa,aA,AA*) which can be found at locus *j* to ((0,0,1),(0,1,0),(1,0,0)). The latter triples indicate which of the three states is present. A genotype of three loci described by (2,0,1) in the numerical GBLUP coding, will here be coded by the nine-tuple (1,0,0,0,0,1,0,1,0) (a triple for each locus, describing its state). We then simply use model Eq. (1) with the new coding. Advantages of this model are that it is also invariant to an exchange of the role of *a* and *A* (as GBLUP of Eq. (1) is as well), since we will only permute the meaning of the positions in the triple but change their entries accordingly. Moreover, we can account for dominance by estimating each effect on its own. A disadvantage is the increased number of variables but this can be overcome easily by the use of relationship matrices for genomic prediction. Property 6 describes the relation between the CM model and GBLUP for markers with only two possible values:

#### **Property 6**

(GBLUP and CM for markers with two possible states) For markers with only two possible states, let **M** denote the *n*×*p* marker matrix in the {−1,1} coding. The relationship matrix of GBLUP is given by (a rescaled version of) **M** **M** ^′^. Moreover, let **C** be the relationship matrix of the CM model. Then

8 $$  \mathbf{C}= 0.5 (\mathbf{MM^{\prime}}+\mathbf{J}_{n\times n} p)  $$

where *p* is the number of markers and **J**_*n*×*n*_ the *n*×*n* matrix with each entry equal to 1.

The linear relationship of the covariance matrices demonstrated in Property 6 implies that the prediction performances of GBLUP and CM are identical for markers with only two possible values.

#### **Property 7**

(Equivalence of GBLUP and CM for markers with two possible states) Let us assume that the ratio of the variance components is fixed such that Property 1 holds for the CM model. Then GBLUP and the CM model are identical for markers with only two possible values.

**A categorical epistasis model (CE)** Analogously to the CM model, we translate the genotype of pairs of loci, e.g. (*aA,bb*) into {0,1}-tuples. Here, a nine-tuple indicates which *combination* of alleles of two loci is present. To translate the genotype (2,0,1) of the numerical {0,1,2} coding into the CE coding, we have to translate each marker pair. Each pair is coded by a nine-tuple with only one entry equal to 1 which indicates the configuration:

9 $$ \left(\underbrace{\bullet}_{(2,2)},\underbrace{\bullet}_{(2,1)},\underbrace{\bullet}_{(2,0)}, \underbrace{\bullet}_{(1,2)},\underbrace{\bullet}_{(1,1)},\underbrace{\bullet}_{(1,0)}, \underbrace{\bullet}_{(0,2)},\underbrace{\bullet}_{(0,1)},\underbrace{\bullet}_{(0,0)}\right).  $$

The assignment of the configuration of the respective marker pair to the position of the nine-tuple can be chosen arbitrarily but has of course to be used consistently for all individuals. Let us assume that we have three subsequent loci with genotypes (2,0,1) in the ordinary numerical coding. Then, there are three possible interactions: the first two loci have the combination (2,0) which will be coded as (0,0,1,0,0,0,0,0,0). Additionally, the second pair is (2,1) which will be coded as (0,1,0,0,0,0,0,0,0), whereas the last pair (0,1) is translated to (0,0,0,0,0,0,0,1,0). As already mentioned, an obvious disadvantage of the model is the high number of variables, but we do not have to solve the system for these variables to perform genomic prediction, since we can use equivalent genomic relationship matrices. Moreover, this model eliminates several disadvantages of EGBLUP: i) The model is invariant with respect to the decision which allele is used as reference (“orientation”), since it is based on categorical variables indicating which genotype is present, ii) the effects the model can assign to different pairs of loci are not connected between pairs by their respective codings (as described for the asymmetrically coded EGBLUP after Example 1), and iii) compared to the symmetric {−1,0,1} coding of EGBLUP, CE does not generally assign the same effects to the most different allele combinations.

### Relationship matrices for the respective marker models

Let **M** be the marker matrix of the respective numerical coding (0,1,2 or −1,0,1). In the following, we will present the corresponding relationship matrices for each model.

**GBLUP.** The relationship matrix for the GBLUP model is given by **M** **M** ^′^ (the *n*×*p* genotype matrix multiplied with its transposed version).

**Epistasis models based on Eq. (** **2** **).** The relationship matrix corresponding to the interactions of Eq. (2) where *j*≥*k* is given by

10 $$  \mathbf{H} = 0.5 \left(\mathbf{MM' \circ MM'}\right) + 0.5 \left(\mathbf{M \circ M}\right) \left(\mathbf{M \circ M}\right)'.  $$

(for a derivation of this statement see [11]). Note here again that the GBLUP model is not affected by a translation of the coding in **M**, but the performance of EGBLUP is affected.

**The categorical marker (CM) effect model** The *i,l*-th entry of the corresponding relationship matrix **C** is given by the inner product of the vectors of the genotypes of individuals *i* and *l* in the coding of the CM model. This means that we count the number of loci which have the same configuration. For markers with two possible variants and the marker data in dosage 0,1 coding, we can express the *i,l*-th entry of **C** the following way:

11 $$  C_{i,l} = p - \sum\limits_{j=1}^{p} \left|M_{i,j} - M_{l,j}\right|  $$

Analogously, for markers with three different variants, we have to count the number of zeros in the marker vectors *M*_*i*,∙_−*M*_*l*,∙_ (For the relation of Eqs. (11) and (8), see the derivation of Eq. (8) in Additional file 2).

**The categorical epistasis (CE) model** The *i,l*-th entry of the corresponding relationship matrix *C*_*E*_ is given by the inner product of the genotypes *i*, *l* in the coding of the categorical epistasis model. Thus, the matrix counts the number of pairs which are in identical configuration and we can express the entry *C*_*E*__*i,l*_ in terms of *C*_*i,l*_ since we can calculate the number of identical pairs from the number of identical loci:

12 $$ {C_{E}}_{i,l}= \sum_{k=1}^{C_{i,l}} k =0.5 C_{i,l} \left(C_{i,l} + 1 \right)  $$

Here, we also count the “pair” of a locus with itself by allowing *k*∈{1,…,*C*_*i,l*_}. Excluding these effects from the matrix would mean, the maximum of *k* equals *C*_*i,l*_−1. In matrix notation Eq. (12) can be written as

13 $$ \mathbf{C}_{E}= 0.5 \mathbf{C} \circ \mathbf{C} + 0.5 \mathbf{C}  $$

Note here, that the relation between GBLUP and the epistasis terms of EGBLUP is identical to the relation of CM and CE in terms of relationship matrices: For **G** **=** **MM** ^′^ and **M** a matrix with entries only 0 or 1, Eq. (10) gives Eq. (13) with **C**=**G** and **C**_*E*_=**H**.

#### **Remark 1**

(The Gaussian kernel) Additionally to the previously discussed EGBLUP model, a common approach to incorporate “non-linearities” is based on Reproducing Kernel Hilbert Space regression [21, 31] by modeling the covariance matrix as a function of a certain distance between the genotypes. The most prominent variant for genomic prediction is the Gaussian kernel. Here, the covariance *Cov*_*i,l*_ of two individuals is described by

$$ Cov_{i,l} = \exp(-b \cdot d_{i,l}), $$

with *d*_*i,l*_ being the squared Euclidean distance of the genotype vectors of individuals *i* and *l*, and *b* a bandwidth parameter that has to be chosen. This approach is independent of translations of the coding, since the Euclidean distance remains unchanged if both genotypes are translated. Moreover, this approach is also invariant with respect to a scaling factor, if the bandwidth parameter is adapted accordingly (in this context see also [32]). Thus, EGBLUP and the Gaussian kernel RKHS approach capture both “non-linearities” but they behave differently if the coding is translated.

### Comparison of the performance of the models on different data sets

**Results on the simulated data** For 20 independently simulated populations of 1 000 individuals, we modeled three scenarios of qualitatively different genetic architecture (purely additive A, purely dominant D and purely epistatic E) with increasing number of involved QTL (see “Methods”) and compared the performances of the considered models on these data. In more detail, we compared GBLUP, a model defined by the epistasis terms of EGBLUP with different codings, the categorical models and the Gaussian kernel with each other. All predictions were based on one relationship matrix only, that is in the case of EGBLUP on the interaction effects only. The use of two relationship matrices did not lead to qualitatively different results (data not shown), but can cause numerical problems for the variance component estimation if both matrices are too similar. For each of the 20 independent simulations of population and phenotypes, test sets of 100 individuals were drawn 200 times independently, and Pearson’s correlation of phenotype and prediction was calculated for each test set and model. The average predictive abilities of the different models across the 20 simulations are summarized in Table 2 in terms of empirical mean of Pearson’s correlation and its average standard error. Comparing GBLUP to EGBLUP with different marker codings, we see that the predictive ability of EGBLUP is very similar to that of GBLUP, if a coding which treats each marker equally is used. Only the EGBLUP version, standardized by subtracting twice the allele frequency as it is done in the commonly used standardization for GBLUP [6], shows a drastically reduced predictive ability for all scenarios (see Table 2, EGBLUP VR). Moreover, considering the categorical models, we see that CE is slightly better than CM and that both categorical models perform better than the other models in the dominance and epistasis scenarios.

**Table 2:** Predictive abilities of the models on the simulated data. Comparison of the predictive abilities in terms of correlations between the measured phenotypes and the predictions for the individuals of the test sets (“Pearson’s correlation”; 100 test set genotypes were drawn randomly from all 1000 genotypes; 200 repeats for each simulated population; 20 independent simulations of population and phenotypes). Traits of different genetic architecture (additive A, dominant D, Epistasis E) and increasing number of QTL. Model abbreviations as introduced in the text. For EGBLUP, only the matrix based on the interactions was considered here

	GBLUP	EGBLUP 0,1,2	EGBLUP -2,-1,0	EGBLUP -1,0,1	EGBLUP VR	CM	CE	K
A1	0.551 ± 0.005	**0.552** ±**0.005**	**0.552** ±**0.005**	0.550 ± 0.005	0.372 ± 0.006	0.489 ± 0.005	0.494 ± 0.005	0.530 ± 0.005
A2	0.549 ± 0.005	**0.550** ±**0.005**	**0.550** ±**0.005**	0.548 ± 0.005	0.351 ± 0.006	0.486 ± 0.005	0.490 ± 0.005	0.527 ± 0.005
A3	0.569 ± 0.005	**0.570** ±**0.005**	**0.570** ±**0.005**	0.568 ± 0.005	0.372 ± 0.006	0.500 ± 0.005	0.504 ± 0.005	0.545 ± 0.005
D1	0.159 ± 0.006	0.160 ± 0.006	0.159 ± 0.006	0.161 ± 0.007	0.111 ± 0.007	0.174 ± 0.006	**0.175** ±**0.006**	0.162 ± 0.006
D2	0.172 ± 0.006	0.172 ± 0.006	0.172 ± 0.006	0.171 ± 0.006	0.103 ± 0.006	**0.186** ±**0.006**	**0.186** ±**0.006**	0.170 ± 0.006
D3	0.156 ± 0.006	0.156 ± 0.006	0.156 ± 0.006	0.158 ± 0.006	0.116 ± 0.006	0.177 ± 0.006	**0.179** ±**0.006**	0.160 ± 0.006
E1	0.244 ± 0.006	0.244 ± 0.006	0.244 ± 0.006	0.244 ± 0.006	0.159 ± 0.006	**0.258** ±**0.006**	**0.258** ±**0.006**	0.243 ± 0.006
E2	0.275 ± 0.006	0.276 ± 0.006	0.276 ± 0.006	0.277 ± 0.006	0.188 ± 0.006	0.301 ± 0.006	**0.302** ±**0.006**	0.277 ± 0.006
E3	0.279 ± 0.006	0.278 ± 0.006	0.279 ± 0.006	0.278 ± 0.006	0.176 ± 0.006	**0.304** ±**0.006**	**0.304** ±**0.006**	0.276 ± 0.006

EGBLUP VR denotes the interaction model based on the by allele frequencies standardized matrix. The given values represent the empirical mean and the corresponding mean standard error across the 20 independently simulated data sets. The highest predictive ability is bold

**Results on the wheat data** For EGBLUP, we used here the coding {0,1} which was originally used in the data of the publication, a translation by −1 which leads to {−1,0} representing a coding in which the meaning of 0 and 1 is permuted, and a centered version {−1,1}. Moreover, we used the standardization by allele frequencies [6] to calculate EGBLUP. Additionally, we evaluated CM, CE and reevaluated the Gaussian kernel RKHS approach, previously used by Crossa et al. [21] (we used the matrix **K** obtained from the supplementary of the corresponding publication). The results are summarized in Table 3. CM showed exactly identical results to those of GBLUP (which has already been stated theoretically by Property 7) and is therefore not listed separately. Considering the predictive ability of EGBLUP with different codings, a first thing to note is that the variability among the EGBLUP variants is higher than that found on the simulated data. Moreover, with the data sets of environments 1,3 and 4, EGBLUP tends to outperform GBLUP. Among them, the model with symmetric {−1,1} coding performs best and the VanRaden standardized version of EGBLUP has a significantly reduced predictive ability for the data of environments 1, 2 and 3, which is analogous to what we have already seen on the simulated data. Moreover, the predictive ability of EGBLUP with symmetric coding seems to be closest to that of the Gaussian kernel. For the data of environment 2, no big differences in the performance of the models (except for the allele frequency standardized EGBLUP) can be observed. Overall, the Gaussian kernel RKHS method performs best on this data set and the predictive ability of the CE model is on the level of the asymmetrically coded versions of EGBLUP.

**Table 3:** Predictive abilities of the models on the wheat data. Comparison of the predictive abilities as Pearson’s correlation of the measured phenotypes and the predictions for the individuals of the test sets (60 test set genotypes, trait: grain yield)

	GBLUP	EGBLUP 0,1	EGBLUP -1,0	EGBLUP -1,1	EGBLUP VR	CE	Gaussian kernel
Environment 1	0.511^*a*^	0.554^*bc*^	0.561^*bcd*^	0.581^*cd*^	0.541^*b*^	0.558^*bcd*^	**0** **.** **5** **8** **4** ^*d*^
Environment 2	0.499^*a*^	0.502^*a*^	**0** **.** **5** **0** **4** ^*a*^	0.495^*a*^	0.422^*b*^	**0** **.** **5** **0** **4** ^*a*^	0.500^*a*^
Environment 3	0.371^*a*^	0.390^*ab*^	0.396^*ab*^	0.409^*b*^	0.365^*a*^	0.393^*ab*^	**0** **.** **4** **2** **2** ^*b*^
Environment 4	0.463^*a*^	0.498^*b*^	0.504^*bc*^	0.530^*c*^	0.500^*b*^	0.502^*b*^	**0** **.** **5** **3** **1** ^*c*^

Letters indicate groups that were not distinguishable at a 5% significance level in a Tukey’s ‘Honest Significant Difference’ test

**Results on the mouse data** We compared the models on 13 traits related to obesity, weight and immunology. Instead of the raw phenotypes, we used pre-corrected residuals which are publicly available (see “Methods”). Again, we compared GBLUP, EGBLUP with 0,1,2 coding as well as with inverted, symmetric and by allele frequencies standardized coding, the categorical models and the Gaussian kernel RKHS approach with each other. The results are summarized in Table 4. The general patterns observed on the previously considered data remain the same: Any EGBLUP version treating the markers equally has at least the same predictive ability as GBLUP for all traits. Among them, the symmetric coding seems to perform best. The allele frequency standardized version of EGBLUP has in three of the 13 traits a higher predictive ability than its other versions (W6W, GrowthSlope, CD8Intensity), but a smaller one in ten cases. Considering only significant differences between CM and GBLUP, CM outperforms GBLUP on the traits %CD4/CD3 and %CD8/CD3 and shows a lower predictive ability only for BMI and BodyLength. Moreover, CE outperforms CM slightly. Overall, two traits are predicted best by EGBLUP VR, three traits by CE, and five by the symmetric version of EGBLUP and the Gaussian kernel, respectively.

**Table 4:** Predictive abilities of the models on the mouse data. Comparison of the predictive abilities as Pearson’s correlation of the measured phenotypes and the predictions for the individuals of the test set (130 test set genotypes). Here, the already for fixed effects pre-corrected residuals of the phenotypes, which are also provided by the publicly available data, were used

	GBLUP	EGBLUP 0,1,2	EGBLUP -2,-1,0	EGBLUP -1,0,1	EGBLUP VR	CM	CE	Gaussian kernel
W6W	0.493^*ab*^	0.540^*c*^	0.505^*ad*^	0.545^*c*^	0.553^*ce*^	0.486^*b*^	0.514^*d*^	**0** **.** **5** **6** **5** ^*e*^
W10W	0.466^*a*^	0.491^*bc*^	0.474^*ab*^	0.495^*bc*^	0.461^*a*^	0.466^*a*^	0.479^*ab*^	**0** **.** **5** **0** **3** ^*c*^
GrowthSlope	0.347^*a*^	0.363^*ab*^	0.350^*a*^	0.364^*ab*^	**0** **.** **3** **7** **5** ^*b*^	0.355^*ab*^	0.363^*ab*^	0.371^*b*^
BMI	0.195^*a*^	0.204^*a*^	0.200^*a*^	**0** **.** **2** **1** **0** ^*a*^	0.194^*a*^	0.153^*b*^	0.166^*b*^	**0** **.** **2** **1** **0** ^*a*^
BodyLength	0.271^*a*^	0.282^*a*^	0.276^*a*^	**0** **.** **2** **8** **5** ^*a*^	0.275^*a*^	0.226^*b*^	0.240^*b*^	0.284^*a*^
%B220	0.549^*ab*^	0.573^*cde*^	0.556^*abc*^	0.576^*de*^	0.540^*a*^	0.547^*ab*^	0.561^*bcd*^	**0** **.** **5** **7** **9** ^*e*^
%CD3	0.522^*a*^	0.535^*a*^	0.527^*a*^	**0** **.** **5** **3** **6** ^*a*^	0.485^*b*^	0.521^*a*^	0.528^*a*^	0.535^*a*^
%CD4	0.495^*a*^	0.506^*a*^	0.499^*a*^	**0** **.** **5** **0** **8** ^*a*^	0.458^*b*^	0.495^*a*^	0.502^*a*^	0.506^*a*^
%CD8	0.694^*a*^	0.703^*ab*^	0.699^*ab*^	0.706^*ab*^	0.656^*c*^	0.706^*ab*^	**0** **.** **7** **1** **1** ^*b*^	0.702^*ab*^
%CD4/CD3	0.643^*a*^	0.655^*abc*^	0.647^*ab*^	0.656^*abc*^	0.618^*d*^	0.660^*bc*^	**0** **.** **6** **6** **4** ^*c*^	0.653^*abc*^
%CD8/CD3	0.683^*a*^	0.689^*ab*^	0.687^*a*^	0.690^*ab*^	0.638^*c*^	0.701^*b*^	**0** **.** **7** **0** **2** ^*b*^	0.686^*a*^
CD4Intensity	0.581^*a*^	0.601^*b*^	0.587^*ab*^	**0** **.** **6** **0** **3** ^*b*^	0.561^*c*^	0.578^*ac*^	0.586^*ab*^	$\mathbf {0.603}^{^{b}}$
CD8Intensity	0.388^*a*^	0.442^*b*^	0.401^*a*^	0.450^*b*^	**0** **.** **4** **8** **1** ^*c*^	0.406^*a*^	0.434^*b*^	0.475^*c*^

Letters indicate groups that were not distinguishable at a 5% significance level in a Tukey’s ‘Honest Significant Difference’ test

For a description of the traits see the corresponding UCL website which is at the moment http://mtweb.cs.ucl.ac.uk/mus/www/mouse/HS/index.shtml

### Incorporating prior experimental information by marker coding

The coding-dependent performance of EGBLUP also offers possibilities to incorporate additional information. He et al. [12,13] have already illustrated the idea of data-driven coding and we have recently shown that information on the performance of genotypes grown under different environmental conditions can be used to select variables within EGBLUP which then can be used for genome assisted prediction within another environment [11]. Here, we will demonstrate that differential coding is also appropriate to incorporate prior experimental information into EGBLUP. For this, we used the different trait (× environment) combinations and adapted the marker coding of each pair of loci to the data, following the procedure described in the “Methods” section. Important here is that we decided for each pair of markers individually, which orientation the corresponding coding of the particular pair shall have. The “orientation” of the underlying effect model is chosen for each pair. Thus, we cut the connection between the coding of different pairs. The determined relationship matrices are then used to predict within the data of other traits. The results are summarized in Tables 5 and 6 for the wheat and mouse data sets, respectively. We can see here that adapting the coding to data of previous experiments can be beneficial for the predictive ability. In the case of the wheat data set, Table 5 shows that using the data of grain yield of the genotypes grown in environments 3 and 4 to infer the marker coding for each pair of marker, improves the prediction accuracy in environment 2 to a level higher than that of all methods which do not use the data of other experiments (from 0.504±0.007 to 0.544±0.006). The situation is analogue for the predictive ability in environment 3, if the data of environment 2 is used to infer the relationship matrix. However, the gain in predictive ability resulting from this procedure is relatively small compared to the gain by means of variable selection [11]. Adapting the coding to given data also helped to increase predictive ability on the mouse data (see Tables 4 and 6). For instance, improvements from 0.285±0.006 to 0.313±0.005, from 0.536±0.004 to 0.569±0.004, and from 0.664±0.004 to 0.685±0.003 were reached for the traits BodyLength, %CD3 and %CD4/CD3, respectively.

**Table 5:** Predictive abilities on the wheat data when prior information is incorporated in the marker coding of EGBLUP. Predictive abilities when the coding for each interaction is determined based on records under different environmental conditions

	G-Env 1	G-Env 2	G-Env 3	G-Env 4
Environment 1	——	0.555 ± 0.007	0.559 ± 0.007	0.552 ± 0.007
Environment 2	0.503 ± 0.007	——	**0.544 ± 0.006**	**0.514 ± 0.007**
Environment 3	0.394 ± 0.008	**0.430 ± 0.008**	——	0.402 ± 0.008
Environment 4	0.500 ± 0.007	0.511 ± 0.006	0.513 ± 0.006	——

G-Env 1 means that the relationship matrix was constructed under the use of the data of Environment 1 (analogously for other environments; for a description of the construction of the matrices see section “Methods”). Bold numbers indicate predictive abilities higher than that of all previously used methods for this trait

**Table 6:** Predictive abilities on the mouse data when prior information is incorporated in the marker coding of EGBLUP. Predictive abilities when the coding for each interaction is determined based on the records of other traits

	G-W6W	G-W10W	G-GrowthSlope	G-BMI	G-BodyLength	G-%B220	
W6W	——	0.548 ± 0.004	0.511 ± 0.004	0.507 ± 0.004	0.511 ± 0.004	0.507 ± 0.004	
W10W	**0.519 ± 0.005**	——	0.480 ± 0.005	0.475 ± 0.005	0.475 ± 0.005	0.474 ± 0.005	
GrowthSlope	0.356 ± 0.005	0.355 ± 0.005	——	0.351 ± 0.005	0.355 ± 0.005	0.351 ± 0.005	
BMI	0.202 ± 0.006	0.202 ± 0.006	0.200 ± 0.006	——	**0.243 ± 0.006**	0.200 ± 0.006	
BodyLength	0.283 ± 0.006	0.278 ± 0.006	0.281 ± 0.006	**0.313 ± 0.005**	——	0.276 ± 0.006	
%B220	0.557 ± 0.004	0.557 ± 0.004	0.557 ± 0.004	0.556 ± 0.004	0.556 ± 0.004	——	
%CD3	0.527 ± 0.004	0.527 ± 0.004	0.527 ± 0.004	0.527 ± 0.004	0.527 ± 0.004	**0.562 ± 0.004**	
%CD4	0.500 ± 0.004	0.500 ± 0.004	0.499 ± 0.004	0.499 ± 0.004	0.500 ± 0.004	**0.530 ± 0.004**	
%CD8	0.701 ± 0.003	0.701 ± 0.003	0.700 ± 0.003	0.700 ± 0.003	0.699 ± 0.003	0.708 ± 0.003	
%CD4/CD3	0.649 ± 0.004	0.649 ± 0.004	0.648 ± 0.004	0.648 ± 0.004	0.647 ± 0.004	0.648 ± 0.004	
%CD8/CD3	0.688 ± 0.003	0.688 ± 0.003	0.687 ± 0.003	0.687 ± 0.003	0.686 ± 0.003	0.687 ± 0.003	
CD4Intensity	0.589 ± 0.004	0.588 ± 0.004	0.588 ± 0.004	0.588 ± 0.004	0.588 ± 0.004	0.588 ± 0.004	
CD8Intensity	0.406 ± 0.005	0.405 ± 0.005	0.404 ± 0.005	0.405 ± 0.005	0.405 ± 0.005	0.404 ± 0.005	
	G-%CD3	G-%CD4	G-%CD8	G-%CD4/CD3	G-%CD8/CD3	G-CD4Intensity	G-CD8Intensity
W6W	0.507 ± 0.005	0.507 ± 0.005	0.507 ± 0.005	0.507 ± 0.005	0.507 ± 0.004	0.507 ± 0.005	0.508 ± 0.005
W10W	0.475 ± 0.005	0.475 ± 0.005	0.475 ± 0.005	0.475 ± 0.005	0.475 ± 0.005	0.475 ± 0.005	0.476 ± 0.005
GrowthSlope	0.351 ± 0.005	0.351 ± 0.005	0.351 ± 0.005	0.351 ± 0.005	0.351 ± 0.005	0.351 ± 0.005	0.351 ± 0.005
BMI	0.200 ± 0.006	0.200 ± 0.006	0.201 ± 0.006	0.201 ± 0.006	0.201 ± 0.006	0.200 ± 0.006	0.202 ± 0.006
BodyLength	0.276 ± 0.006	0.276 ± 0.006	0.276 ± 0.006	0.276 ± 0.006	0.276 ± 0.006	0.276 ± 0.006	0.277 ± 0.006
%B220	**0.588 ± 0.004**	**0.582 ± 0.004**	0.570 ± 0.004	0.557 ± 0.004	0.557 ± 0.004	0.556 ± 0.004	0.558 ± 0.004
%CD3	——	**0.569 ± 0.004**	**0.550 ± 0.004**	0.527 ± 0.004	0.527 ± 0.004	0.527 ± 0.004	0.527 ± 0.004
%CD4	**0.545 ± 0.004**	——	0.504 ± 0.004	**0.511 ± 0.004**	**0.510 ± 0.004**	0.500 ± 0.004	0.499 ± 0.004
%CD8	**0.714 ± 0.003**	0.702 ± 0.003	——	**0.722 ± 0.003**	**0.726 ± 0.003**	0.700 ± 0.003	0.7 ± 0.003
%CD4/CD3	0.649 ± 0.004	0.656 ± 0.004	**0.672 ± 0.004**	——	**0.685 ± 0.003**	0.649 ± 0.004	0.649 ± 0.004
%CD8/CD3	0.688 ± 0.003	0.694 ± 0.003	**0.714 ± 0.003**	**0.721 ± 0.003**	——	0.687 ± 0.003	0.687 ± 0.003
CD4Intensity	0.588 ± 0.004	0.589 ± 0.004	0.589 ± 0.004	0.589 ± 0.004	0.588 ± 0.004	——	0.595 ± 0.004
CD8Intensity	0.403 ± 0.005	0.403 ± 0.005	0.403 ± 0.005	0.405 ± 0.005	0.404 ± 0.005	0.414 ± 0.005	——

G-W6W means that the relationship matrix was constructed under the use of the pre-corrected residuals of the trait W6W. Bold numbers indicate predictive abilities higher than that of all previously used methods for this trait

## Discussion

### The effect of the choice of marker coding on EGBLUP

We recalled that GBLUP is not sensitive to certain changes of the marker coding if the variance components are adapted accordingly. Analogously, we also proved that the interaction terms of EGBLUP are invariant to factors rescaling the marker coding, but showed that a translation indeed changes the underlying marker effect model drastically. In particular, we demonstrated that the effect model of EGBLUP with the asymmetric 0,1,2 coding is affected by the decision which allele to count. Thus, an important observation concerning EGBLUP is that the only coding allowing a permutation of the roles of the alleles without changing the underlying interaction effect model for the respective marker pair is symmetric around zero. This coding solves the problem of “which allele to count”, but we also argued that the symmetric coding appears to be biologically implausible since it assigns the same interaction effect to the most distant genotypes. Concerning the allele frequency adjusted version EGBLUP VR, we illustrated that the different markers are not treated equally and thus that the interaction effect models here depend on the allele frequencies of the involved alleles. On the level of predictive ability, the symmetric coding tends to outperform the asymmetric versions slightly, which can most clearly be seen from the data of environment 1 and 4 of the wheat data set (Table 3). Also with the mouse data set, the symmetric coding had a higher predictive ability than the other codings treating all loci equally for all traits, but the improvements were most often very small. Concerning the allele-frequencies standardized version EGBLUP VR, we observed a drastic reduction in the predictive ability compared to other EGBLUP versions in most of the examples. Illustratively, one reason for the comparatively poor performance can be seen in the following: the relationship matrix corresponding to the interaction effects of EGBLUP in a certain coding is basically the GBLUP relationship matrix, but with each of its entries squared (if all pairwise interactions and interactions of a marker with itself are modeled, see [10,11] and compare to Eq. (10)). The standardization by twice the allele frequencies (and division by a certain factor representing a variance [6]) produces a GBLUP matrix which can possess entries larger than 1 and smaller than 0. In particular, if the GBLUP matrix has negative entries, squaring them changes the order of the relationship between the individuals. For instance, if A has a relation of −0.1 with individual *B* and −0.3 with individual *C*, which means that A is more closely related to *B* than to *C*, the corresponding EGBLUP matrix states that the relation between *A* and *C* is closer than that of *A* and *B*. This argumentation is equally true for the symmetric coding, but the portion of negative entries in the corresponding additive relationship matrix was close to zero for the wheat and the mouse data set when the symmetric coding was used in our examples. Overall, in spite of a certain popularity of EGBLUP in recent literature [10,11,17] our results suggest that the use of products of marker values as predictor variables is not the best way to incorporate interactions into the GBLUP model. Moreover, contrary to the theoretical findings on the “congruency” of EGBLUP and the Gaussian kernel in a RKHS approach [10], our results show that both methods respond in a different way to a change of marker coding: a translation of the coding has an impact on the predictive ability of EGBLUP, but not on that of the Gaussian kernel. Since the Euclidean distance between two vectors will not change under a translation of both vectors, the corresponding relationship matrix remains identical. A reconsideration of the limit behavior of EGBLUP when the degree of interaction increases to *n*-factor interaction (and *n*→*∞*) may therefore be interesting from a theoretical point of view.

### Categorical effect models

To develop an alternative to EGBLUP which does not possess the illustrated undesired theoretical properties, but which –unlike the RKHS approaches– allows to interpret the predicted quantities as “effects”, we considered the categorical effect models (The effects of the categorical models can be explicitly calculated from phenotypes or genetic values under the use of the well-known Mixed Model formulas for effects with the respective design matrices). As a first step, we constructed the categorical marker effect model CM, which does not use the assumption of a constant allele substitution effect (Fig. 1) and thus gives the possibility to model (over)dominance by modeling an independent effect of each genotype at a locus. The fact that this property can also lead to an increase in predictive ability was illustrated by the simulated dominance scenario. An important result is that this categorical model can be rewritten as a relationship matrix model and thus provides an equivalent to the Ridge Regression/GBLUP duality, but based on a categorical effect model instead of a numerical dosage model. Whether this model increases predictive ability will always depend on the population structure and the influence of dominance effects on a particular trait. For instance, if a population originating from lines from different heterotic pools is considered, the prevalent heterosis effect might be a good reason to use CM instead of GBLUP, since heterosis creates a deviation from the linear dosage model. Moreover, the number of heterozygous and homozygous loci in the data set is important. If most loci are mainly present in only two of the three possible SNP genotypes, CM cannot outperform GBLUP substantially. Interestingly, comparing GBLUP and CM, CM was only significantly outperformed on the traits BMI and BodyLength. Thus, abandoning the assumption of a dosage effect of an allele, which is implemented by counting its occurrence and multiplying it with an additive effect, might not in general be a problem for prediction. Note also that there are other ways of defining marker based dominance matrices as for instance described by Su et al. [33]. Moreover, dominance can implicitly be modeled by an epistatic interaction term of a locus with itsself in Eq. (2) if *j*=*k* (see [11]).

Analogously to the relation of GBLUP and EGBLUP, we extended the categorical marker effect model CM to the categorical epistasis model CE. The disadvantage of inflating the model with a huge number of variables is solved for genomic prediction by using an equivalent relationship-matrix-based approach. Interestingly, the analogy of the relation between GBLUP and EGBLUP also translates to the level of relationship matrices, which we illustrated by the theoretical result of Eq. (13). The relationship matrix of CE has the same connection to the relationship matrix of CM as the matrix defined by the interaction terms of EGBLUP has to the genomic relationship matrix of GBLUP. Moreover, CE eliminates undesired theoretical properties of EGBLUP: the question which allele to use as reference is not raised, its structure does not lead to a dependence of the effect models of different pairs of loci, and it does not assign the same effects to the most different allele combinations as the symmetrically coded EGBLUP model does. With the wheat data which consist of markers with only two possible values and for which GBLUP coincides with CM, CE outperformed GBLUP in all environments (Table 3). Moreover, CE slightly improved the predictive ability of CM for all considered traits of the mouse data set. Overall, the CE model is a valuable alternative for modeling epistasis since it eliminates undesired properties of EGBLUP and shows convincing results in practice. However, other more realistic parametric structures of effects in between EGBLUP and CE may be of interest for future research. Important steps into this direction have already been made with the “hybrid” coding according to He et al. [12,13], in which the marker coding is estimated from the data under the side condition of generating a monotone effect model. Moreover, an interesting approach for future investigation may be the adaption of categorical models to other types of variables, for instance defined by haplotypes.

### Incorporating prior experimental information into the coding of EGBLUP

Finally, we demonstrated that marker coding can be used to incorporate prior information. An important property of the procedure we used is that we “decoupled” the effect models for different pairs by allowing to choose the orientation of the parametric model for each pair separately (see “Methods”). In particular, this means that marker *j* might be coded as 0,1,2 in combination with marker *k*, but as −2,−1,0 in combination with marker *l*. The criterion to decide which coding to use, was simple here by comparing the size of the absolute interaction effect of a pair when different “orientations” were used. Note here that the improvement of prediction accuracy was smaller than by means of variable selection on the wheat data set [11]. The relatively small improvement might be a result of only giving the two possibilities of both markers being in the initial coding or both markers with inverted coding, but not choosing from all possible four orientations. We used this simplified procedure, since for other combinations of one marker with original coding and the other marker with inverted coding, the assigned effect will also depend on the orientation of other pairs and thus it is difficult to determine which orientation to choose if we will additionally change the orientation of other pairs. In this regard, the presented method can be considered as a straightforward ad hoc approach to incorporate prior knowledge into the coding, capturing some part of the covariance structure of the given data and thus improving the predicitve ability on data sets with similar covariance structure.

## Conclusion

We illustrated that the EGBLUP model possesses several undesired properties caused by the interactions being modeled by products of marker values. We showed that the symmetrically coded EGBLUP tends to perform best, that the allele frequency standardized version tends to have the lowest predicitve ability and that the CE model can be an attractive alternative to EGBLUP. Prior information from other experiments can be incorporated into the marker coding of EGBLUP, which gives the potential to enhance predictive ability for correlated traits.

## Endnote

^1^ In literature, the expression GBLUP is used for the reformulated equivalent of Eq. (1) with genetic value **g**:=**M** ***β*** and thus $\mathbf {g}\sim \mathcal {N}(0,\sigma ^{2}_{\beta } \mathbf {MM}')$.

## Additional files

Additional file 1 Rdata-file with two lists. The list “Mouse_Data” contains a genotype matrix of 1298 individuals and 9265 markers as well as a matrix with records of 13 traits of the individuals. The list “Simulated_Data” offers the genotypes and phenotypes of the 20 simulations. Each entry of this list is a list of two elements representing genotypes and phenotypes of the respective simulation. Genotypes are given by a matrix of 1000 individuals with 9000 markers. Phenotypes are provided as a data.frame of the 1000 individuals and the 9 different phenotypes described in the Methods section. (RDATA 64512 kb)

Additional file 2 The file presents mathematical arguments for the statements on the properties of the models, which have been made in the main text. (PDF 149 kb)

## Abbreviations

CM: Categorical marker effect model
CE: Categorical epistais model
DArT: Diversity Arrays Technology
EGBLUP: Extended genomic best linear unbiased prediction
GBLUP: Genomic best linear unbiased prediction
MAF: Minor allele frequency
SNP: Single nucleotide polymorphism

## References

[1] Meuwissen T, Hayes B, Goddard M (2001). Prediction of total genetic value using genome-wide dense marker maps. Genetics.

[2] Hayes BJ, Visscher PM, Goddard ME (2009). Increased accuracy of artificial selection by using the realized relationship matrix. Genet Res.

[3] Abraham G, Tye-Din JA, Bhalala OG, Kowalczyk A, Zobel J, Inouye M (2014). Accurate and robust genomic prediction of celiac disease using statistical learning. PLoS Genet.

[4] Henderson CR (1975). Best linear unbiased estimation and prediction under a selection model. Biometrics.

[5] Habier D, Fernando R, Dekkers J (2007). The impact of genetic relationship information on genome-assisted breeding values. Genetics.

[6] VanRaden P (2008). Efficient methods to compute genomic predictions. J Dairy Sci.

[7] Piepho HP (2009). Ridge regression and extensions for genomewide selection in maize. Crop Sci.

[8] Albrecht T, Wimmer V, Auinger HJ, Erbe M, Knaak C, Ouzunova M, Simianer H, Schön CC (2011). Genome-based prediction of testcross values in maize. Theor Appl Genet.

[9] Strandén I, Christensen OF (2011). Allele coding in genomic evaluation. Genet Sel Evol.

[10] Jiang Y, Reif JC (2015). Modeling epistasis in genomic selection. Genetics.

[11] Martini JWR, Wimmer V, Erbe M, Simianer H (2016). Epistasis and covariance: How gene interaction translates into genomic relationship. Theor Appl Genet.

[12] He D, Wang Z, Parida L (2015). Data-driven encoding for quantitative genetic trait prediction. BMC Bioinformatics.

[13] He D, Parida L (2016). Does encoding matter? a novel view on the quantitative genetic trait prediction problem. BMC Bioinformatics.

[14] Falconer DS, Mackay TF, Frankham R. Introduction to quantitative genetics. 10.1093/genetics/167.4.1529 PMC1471025 15342495

[15] Zeng ZB, Wang T, Zou W (2005). Modeling quantitative trait loci and interpretation of models. Genetics.

[16] Hallgrímsdóttir IB, Yuster DS (2008). A complete classification of epistatic two-locus models. BMC Genet.

[17] Hu Z, Li Y, Song X, Han Y, Cai X, Xu S, Li W (2011). Genomic value prediction for quantitative traits under the epistatic model. BMC Genet.

[18] Mackay TF (2014). Epistasis and quantitative traits: using model organisms to study gene-gene interactions. Nat Rev Genet.

[19] Wang D, El-Basyoni IS, Baenziger PS, Crossa J, Eskridge K, Dweikat I (2012). Prediction of genetic values of quantitative traits with epistatic effects in plant breeding populations. Heredity.

[20] Sargolzaei M, Schenkel FS (2009). QMSim: a large-scale genome simulator for livestock. Bioinformatics.

[21] Crossa J, de Los Campos G, Pérez P, Gianola D, Burgueno J, Araus JL, Makumbi D, Singh RP, Dreisigacker S, Yan J, Arief V, Banziger M, HJ B (2010). Prediction of genetic values of quantitative traits in plant breeding using pedigree and molecular markers. Genetics.

[22] Solberg LC, Valdar W, Gauguier D, Nunez G, Taylor A, Burnett S, Arboledas-Hita C, Hernandez-Pliego P, Davidson S, Burns P (2006). A protocol for high-throughput phenotyping, suitable for quantitative trait analysis in mice. Mamm Genome.

[23] Valdar W, Solberg LC, Gauguier D, Cookson WO, Rawlins JNP, Mott R, Flint J (2006). Genetic and environmental effects on complex traits in mice. Genetics.

[24] Durinck S, Spellman PT, Birney E, Huber W (2009). Mapping identifiers for the integration of genomic datasets with the r/bioconductor package biomart. Nat Protoc.

[25] Durinck S, Moreau Y, Kasprzyk A, Davis S, De Moor B, Brazma A, Huber W (2005). Biomart and bioconductor: a powerful link between biological databases and microarray data analysis. Bioinformatics.

[26] Wimmer V, Albrecht T, Auinger HJ, Schoen CC (2012). synbreed: a framework for the analysis of genomic prediction data using R. Bioinformatics.

[27] Akdemir D, Godfrey OU. EMMREML: Fitting Mixed Models with Known Covariance Structures. 2015. R package version 3.1. http://CRAN.R-project.org/package=EMMREML.

[28] R Core Team (2014). R: A Language and Environment for Statistical Computing.

[29] Ober U, Huang W, Magwire M, Schlather M, Simianer H, Mackay TF (2015). Accounting for genetic architecture improves sequence based genomic prediction for a drosophila fitness trait. PloS ONE.

[30] Zhang Z, Ober U, Erbe M, Zhang H, Gao N, He J, Li J, Simianer H (2014). Improving the accuracy of whole genome prediction for complex traits using the results of genome wide association studies. PloS ONE.

[31] Gianola D, Morota G, Crossa J. Genome-enabled prediction of complex traits with kernel methods: What have we learned? In: Proceedings of the 10th World Congress of Genetics Applied to Livestock Production. Vancouver, BC, Canada: 2014. https://asas.confex.com/asas/WCGALP14/webprogram/Paper10331.html.

[32] Long N, Gianola D, Rosa GJ, Weigel KA (2011). Marker-assisted prediction of non-additive genetic values. Genetica.

[33] Su G, Christensen OF, Ostersen T, Henryon M, Lund MS (2012). Estimating additive and non-additive genetic variances and predicting genetic merits using genome-wide dense single nucleotide polymorphism markers. PloS ONE.

