# Supplementary material for: Genomic prediction with epistasis models: on the marker-coding-dependent performance of the extended GBLUP and properties of the categorical epistasis model (CE)
Source: BMC Bioinformatics. 2017 Jan 3;18:3. doi: 10.1186/s12859-016-1439-1 (PMC5209948; doi:10.1186/s12859-016-1439-1)
Supplement: Additional file 2 — The file presents mathematical arguments for the statements on the properties of the models, which have been made in the main text. (PDF 149 kb) [file 12859_2016_1439_MOESM2_ESM.pdf]

Genomic prediction with epistasis models:
On the marker-coding-dependent performance of the extended GBLUP and properties of the categorical epistasis model (CE)
# – Appendix –

In this section, we will give short mathematical proofs for the statements made in the main text.

Proof 1 (Property 1) *The standard approach for the estimation / prediction of the parameters of the mixed model is to maximize the joint density of phenotypes* $\mathbf{y}$ *and the additive effects* $\boldsymbol{\beta}$ *(conditioned on the fixed effect* μ*; multivariate Gaussian, product of the density of* $\boldsymbol{\beta}$ *and the density of the conditional distribution of* $\mathbf{y}$ *for fixed* $\boldsymbol{\beta}$*; the variance components are usually assumed to be known) with respect to* μ *and* $\boldsymbol{\beta}$ *[4]. This approach leads to the linear system*

$$\begin{pmatrix} \mathbf{1}'\mathbf{1} & \mathbf{1}'\mathbf{M} \\ \mathbf{M}'\mathbf{1} & \mathbf{M}'\mathbf{M}+\mathbf{I}_p\frac{\sigma_\varepsilon^2}{\sigma_\beta^2} \end{pmatrix} \begin{pmatrix} \hat{\mu} \\ \hat{\beta} \end{pmatrix} = \begin{pmatrix} \mathbf{1}'\mathbf{y} \\ \mathbf{M}'\mathbf{y} \end{pmatrix}. \tag{14}$$

Here, $\mathbf{1}$ *denotes the* $n \times 1$ *vector with all entries equal to* 1*,* $\mathbf{M}$ *is the matrix of genotypes,* $\mathbf{I}_p$ *is the p-dimensional identity matrix and* σ_i^2 *is the respective variance component of the independent Gaussian random terms* $\boldsymbol{\epsilon}$ *or* $\boldsymbol{\beta}$ *(recall Eq. (1) for the model description). What we have to show to prove a) and b) is that* $\hat{\mu}, \hat{\beta}$ *solving system (14) implies that* $\tilde{\mu} := \hat{\mu} + \mathbf{P}'\hat{\beta}$*,* $\hat{\beta}$ *solve the system*

$$\begin{pmatrix} \mathbf{1}'\mathbf{1} & \mathbf{1}'(\mathbf{M}-\mathbf{1}\mathbf{P}') \\ (\mathbf{M}-\mathbf{1}\mathbf{P}')'\mathbf{1} & (\mathbf{M}-\mathbf{1}\mathbf{P}')'(\mathbf{M}-\mathbf{1}\mathbf{P}')+\mathbf{I}_p\frac{\sigma_\varepsilon^2}{\sigma_\beta^2} \end{pmatrix} \begin{pmatrix} \tilde{\mu} \\ \hat{\beta} \end{pmatrix} = \begin{pmatrix} \mathbf{1}'\mathbf{y} \\ (\mathbf{M}-\mathbf{1}\mathbf{P}')'\mathbf{y} \end{pmatrix}, \tag{15}$$

which can be verified by a calculation. Statement c) is a consequence of the predicted average phenotype being $\tilde{\mathbf{y}} = \mathbf{1}\tilde{\mu} + \tilde{\mathbf{M}}\tilde{\beta}$*.*

Proof 2 (Property 2) *Analogously to the proof of Property 1, substitute* $\mathbf{M}$*,* σ_β^2 *and* $\hat{\beta}$ *by* $c\mathbf{M}$*,* $c^{-2}\sigma_\beta^2$ *and* $c^{-1}\hat{\beta}$*.*

Proof 3 (Property 3) *Analogously to the proof of Property 1, we maximize the joint density of* $\mathbf{y}, \boldsymbol{\beta}, \boldsymbol{h}$ *(conditioned on the fixed effect* μ*) with respect to* μ*,* $\boldsymbol{\beta}$ *and* $\boldsymbol{h}$*. Thus, we have to find a local extreme of*

$$(\mathbf{y}-\mathbf{1}\mu-\mathbf{M}\beta-\mathbf{N}\mathbf{h})'\frac{1}{\sigma_\varepsilon^2}\mathbf{I_n}(\mathbf{y}-\mathbf{1}\mu-\mathbf{M}\beta-\mathbf{N}\mathbf{h})+\beta'\frac{1}{\sigma_\beta^2}\mathbf{I_p}\beta+\boldsymbol{h}'\frac{1}{\sigma_h^2}\mathbf{I}_{\ell(\boldsymbol{N})}\boldsymbol{h}. \tag{16}$$

All variables are as previously defined, with $\boldsymbol{h}$ additionally denoting the vector of all interactions and $\mathbf{N}$ denoting the $n \times \ell(N)$ matrix assigning the respective products of marker values of each of the n individuals to the respective interaction. The length $\ell(N)$ of the rows of matrix N depends on how many interactions are incorporated in the model (e.g. $\ell(N) = p^2$). The important fact is that each entry of $\mathbf{N}$ is a product of two marker values. This implies that if we change $\mathbf{M}$ to $c\mathbf{M}$, we change $\mathbf{N}$ to $c^2\mathbf{N}$. Calculating the partial derivatives of Eq. (16) with respect to μ, $\boldsymbol{\beta}$ and $\boldsymbol{h}$ gives the linear system

$$\begin{pmatrix} \mathbf{1}'\mathbf{1} & \mathbf{1}'\mathbf{M} & \mathbf{1}'\mathbf{N} \\ \mathbf{M}'\mathbf{1} & \mathbf{M}'\mathbf{M} + \mathbf{I_p}\frac{\sigma_\varepsilon^2}{\sigma_\beta^2} & \mathbf{M}'\mathbf{N} \\ \mathbf{N}'\mathbf{1} & \mathbf{N}'\mathbf{M} & \mathbf{N}'\mathbf{N} + \mathbf{I}_{\ell(N)}\frac{\sigma_\varepsilon^2}{\sigma_h^2} \end{pmatrix} \begin{pmatrix} \hat{\mu} \\ \hat{\boldsymbol{\beta}} \\ \hat{\boldsymbol{h}} \end{pmatrix} = \begin{pmatrix} \mathbf{1}'\mathbf{y} \\ \mathbf{M}'\mathbf{y} \\ \mathbf{N}'\mathbf{y} \end{pmatrix}. \tag{17}$$

If we substitute here $\mathbf{M}$ by $c\mathbf{M}$, $\mathbf{N}$ by $c^2\mathbf{N}$, σ_β^2 by $c^{-2}\sigma_\beta^2$ and σ_h^2 by $c^{-4}\sigma_h^2$, the new system will be solved by $\tilde{\mu}$, $\tilde{\boldsymbol{\beta}}$ and $\tilde{\boldsymbol{h}}$ as stated.

Proof 4 (Property 4) *Let loci k and j share the same coding. The weights assigned to certain allele combinations are then described by*

$$\begin{array}{cccc} & aa & aA & AA \\ bb & M_{aa}^2 & M_{aa}M_{aA} & M_{aa}M_{AA} \\ bB & M_{aa}M_{aA} & M_{aA}^2 & M_{aA}M_{AA} \\ BB & M_{aa}M_{AA} & M_{aA}M_{AA} & M_{AA}^2 \end{array} \tag{18}$$

If we permute the role of a and A, we mirror the matrix with respect to the middle column. This means, if the model shall be invariant, instead of $\hat{h}_{j,k}$ we should estimate another $\tilde{h}_{j,k}$ such that the former model multiplied with $\hat{h}_{j,k}$ equals the new coding multiplied with $\tilde{h}_{j,k}$. This has to be possible for any $\hat{h}_{j,k}$, in particular for $\hat{h}_{j,k} \neq 0$, and thus $\tilde{h}_{j,k} \neq 0$ (otherwise the effects of the two models cannot be equal). Consequently, a constant $c := \frac{\hat{h}_{j,k}}{\tilde{h}_{j,k}}$ such that the former matrix of weights multiplied by c equals the new weights. In particular this means that the initial weight for $(bb, aa) = M_{aa}^2$ multiplied with c equals the new weight $M_{aa}M_{AA}$ and the initial weight for $(bb, AA) = M_{aa}M_{AA}$ multiplied by c equals the weight M_{aa}^2:

$$cM_{aa}^2 = M_{aa}M_{AA} \qquad \text{and} \qquad cM_{aa}M_{AA} = M_{aa}^2. \tag{19}$$

If $M_{aa} \neq 0$, we have $c^2 = 1$ and thus $c = \pm 1$. If $c = 1$, $M_{aa} = M_{AA}$ which is not allowed, since we are only considering codings with three different values for aa, aA, AA. Then $c = -1$ implies that $M_{aA} = 0$, since $-M_{aa}M_{aA} = M_{aa}M_{aA}$, and that $-M_{aa} = M_{AA}$, since $-M_{aa}^2 = M_{aa}M_{AA}$.

If $M_{aa} = 0$, consider the second row of matrix (18). The reasoning described above gives $cM_{aa}M_{aA} = M_{aA}M_{AA}$, which would imply that $M_{aA} = 0$ or $M_{AA} = 0$, which is not possible since we want to code the three allele combinations differently. Thus, $M_{aa} = 0$ is a contradiction to the model being invariant with respect to the decision which allele to count. Analogously for markers with only two possible values.

Proof 5 (Property 5) *Let us choose three products of Eq. (7) such that variables M_{aa}, M_{aA}, M_{AA} are included as a factor of at least one product. i) If we fix the diagonal $\{a_{m,m}\}_{m=1}^{3}$, the marker values are given as the square roots (possibly as a complex number with imaginary part nonzero). ii) Let us choose two products on the diagonal and one other product of two different variables (one of them shall not be included in the products on the diagonal). Then the square roots of the elements on the diagonal determine two variables and the remaining variable can be calculated from the last product. iii) Let us choose one element on the diagonal and two elements off-diagonal. Then the corresponding marker value of the diagonal element is determined. One of the other products $a_{r,s}$ is the product of the same variable and another marker value, which determines the other marker value. Analogously for the last variable. iv) Let us choose the three off-diagonal elements. Then we have to solve the system*

$$a_{1,2} = M_{aa}M_{aA} \qquad a_{1,3} = M_{aa}M_{AA} \qquad a_{2,3} = M_{aA}M_{AA},$$

which has a unique solution (up to a sign).

Proof 6 (Property 6) *The (i,l)-th entry of $\mathbf{MM}'$ in the $\{-1, 1\}$ coding counts the number of loci in which individual i and l have the same marker value (this is equal to $C_{i,l}$) and subtracts the number of loci with different configuration ($p - C_{i,l}$). Thus $(MM')_{i,l} = 2C_{i,l} - p$.*

Proof 7 (Property 7) *Let $\mathbf{Q}$ denote the $n \times 2p$ matrix giving the coding of the CM model for the n individuals (recall here that we are considering markers with only two variants and $\mathbf{QQ}' = \mathbf{C}$ of Property 6). We know that the marker effect model is equivalent to a model with a corresponding relationship matrix. Moreover, we know from Property 1 that the model is independent of translations of the coding, since it is an additive model. Consequently, it is enough to show that a rescaled version of the GBLUP relationship matrix $\mathbf{MM}'$ is identical to the relationship matrix defined by a translation of $\mathbf{Q}$. This means that we have to*

show that $\alpha \in \mathbb{R}$ *and a* $2p \times 1$ *vector* $\mathbf{P}$ *exist such that*

$$\mathbf{MM}' = \alpha(\mathbf{Q} - \mathbf{1P}')(\mathbf{Q} - \mathbf{1P}')'.$$

Since the rowsum of $\mathbf{Q}$ *equals the number of markers* p *for every row and due to the statement of Proposition 6, this equation is satisfied if* $\alpha = 2$ *and the vector* $\mathbf{P}$ *has the constant entry* 0.5.
